# A New Remedy in Amenorrhœa and Dysmenorrhœa

**Published:** 1891-02

**Authors:** F. S. Mason

**Affiliations:** Pharmacist


### Selections and Abstracts

### A NEW REMEDY IN AMENORRHŒA AND DYSMENORRHŒA.

Various methods for the extraction of the active principle of Parsley have been proposed from time to time, but there has been always a want of uniformity in the therapeutic results obtained with the so-called Apiol preparations, hitherto found in commerce.

With a view to obtain a reliable product, M. Chapoteaut recommenced a study of the plant and finally adopted a new process for the extraction of thick reddish liquid boiling at 275 deg. C. (527 deg. F.) specific gravity 1.113.

This is a product totally different from true Apiol, since this latter is a solid melting at 30 deg. and boiling at 300 deg. C., and different from the Essence or Oil of Parsley, boiling at 160 deg. C.; while its reddish color indicates that it cannot be confounded with ordinary so-called commercial Apiol, which is a yellow or green liquid having an approximate specific gravity of 1.07.

This new substance therefore has been named Apioline (Apiolinum) by M. Chapoteaut, and clinical experiments show it to be the true active principle of the plant.

Dr. Laborde, Director of the Physiological Department of Faculty (Paris) gives an ethaustive report on the action of this drug and its derivatives, cariol, etc., on guinea pigs and dogs (see Tribune Medicale, Jan. 1891) from which experiments it appears that Apioline stimulates the circulatory system of the intestines and genitals, causing vascular congestion of the uterus and ovaries and exciting contraction of the smooth muscular fibres of the genital organs, especially of the uterus.

These results have been remarkably confirmed by their therapeutic application in the French Hospitals.

Apioline–Chapoteaut administered in spherical capsules of 20 centigrammes each, always relieved the pain in spasmodic and congestive Dysmenorrhœa, cases in which principle reliance should be placed on equalizing the circulation and increasing the power of the ovarian nisus.

In Amenorrhœa, where the menses had been suppressed even for a considerable length of time, the flow promptly re-appeared.

In fact, in all cases depending on uterine troubles amenable to internal treatment, and where a correct diagnostic of the symptoms had been made and suitable hygiene and treatment observed, this drug relieved the suppression, regulated and prevented or removed the accompanying pain, and proved to be the most powerful emmenagogue with which we are familiar.

In cases of scanty or deficient menstruation with pains, etc., one capsule can be given after meals, thrice daily for a week before the expected period, as recommended by Dr. Fordyce Barker.*

℞
Apiolini grm. IV. (about ℥ j).
ft. Capsulæ No. XX (Chapoteaut).

Sig: Take three each day during the week preceding menstruation.

It is especially appropriate when amenorrhœa depends upon anæmia. The same authority suggests the administration of Aloine or podophyllotoxin when amenorrhœa and dysmenorrhœa are complicated with constipation. Although Apioline is looked on as a specific for menstrual disorders by many gynœcologists, it must not be forgotten that these troubles are often subordinate or associated with a general atony of the system, which require tonics, hœmatics (Ferrum Sanguinis) and suitable hygienic agents. Finally Apioline-Chapoteaut cannot be expected to remove dysmenorrhœa depending on mechanical obstruction of the cervical canal—causes of failure which are sometimes overlooked.

F. S. MASON, Pharmacist.

